# Erythropoietin enhances Kupffer cell number and activity in the challenged liver

**DOI:** 10.1038/s41598-017-11082-7

**Published:** 2017-09-04

**Authors:** Dafna Gilboa, Yasmin Haim-Ohana, Naamit Deshet-Unger, Nathalie Ben-Califa, Sahar Hiram-Bab, Debby Reuveni, Ehud Zigmond, Max Gassmann, Yankel Gabet, Chen Varol, Drorit Neumann

**Affiliations:** 1 Department of Cell and Developmental Biology, Sackler Faculty of Medicine, Tel Aviv University, Tel Aviv, Israel; 2 Department of Anatomy and Anthropology, Sackler Faculty of Medicine, Tel Aviv University, Tel Aviv, Israel; 3 The Research Center for Digestive Tract and Liver Diseases, Sourasky Medical Center and Department of Clinical Microbiology and Immunology, Sackler Faculty of Medicine, Tel Aviv University, Tel Aviv, Israel; 4 Institute for Veterinary Physiology, Vetsuisse Faculty and Zurich Center for Integrative Human Physiology (ZIHP), University of Zurich, Zurich, Switzerland

## Abstract

Erythropoietin (EPO) is the main hormone driving mammalian erythropoiesis, with activity mediated *via* the surface receptor, EPO-R, on erythroid progenitor cells. Recombinant human EPO is currently used clinically for the treatment of anemia in patients with end-stage renal disease, and in certain cancer patients suffering from anemia induced either by the tumor itself or by chemotherapy. EPO-R expression is also detected in non-erythroid cells, including macrophages present in the peritoneum, spleen, and bone marrow (BM). Here we demonstrate that Kupffer cells (KCs) - the liver-resident macrophages - are EPO targets. We show that, *in vitro*, EPO initiated intracellular signalling and enhanced phagocytosis in a rat KC line (RKC-2) and in sorted KCs. Moreover, continuous EPO administration in mice, resulted in an increased number of KCs, up-regulation of liver EPO-R expression and ﻿﻿﻿elevated﻿ production of the monocyte chemoattractant CCL2, with corresponding egress of Ly6C^hi^ monocytes from the BM. In a model of acute acetaminophen-induced liver injury, EPO administration increased the recruitment of Ly6C^hi^ monocytes and neutrophils to the liver. Taken together, our results reveal a new role for EPO in stimulating KC proliferation and phagocytosis, and in recruiting Ly6C^hi^ monocytes in response to liver injury.

## Introduction

Erythropoietin (EPO) is the main hormone that drives mammalian erythropoiesis in response to hypoxia, through activation of the hypoxia inducible factor HIF-2^1^. It is synthesized by the adult kidney and by the fetal liver as well as by the adult liver^2, 3^ and regulates the production of red blood cells *via* its receptor, EPO-R. Recombinant human EPO (rHuEPO) is currently used in clinical applications for the treatment of anemia associated with chronic renal failure^4, 5^, as well as for alleviating chemotherapy or radiotherapy-induced anemia in certain cancer patients^6^. However, it has become clear that the indications that can benefit from EPO treatment extend beyond anemia^7–15^. EPO treatment has been found to have a tissue-protective effect in animal models reflecting a wide variety of tissues. Among others, the beneficial effects of EPO have been reported in various models of liver injuries such as fibrosis, ischemia/reperfusion (I/R) injury, and extended liver resection^16–18^. In addition, the combination of G-CSF (Granulocyte Colony-Stimulating Factor) and Darbepoetin α, an EPO derivative with prolonged serum half-life, provided clinical benefit and improved survival in patients with decompensated liver disease^19^.

The liver is a unique immunological organ and one of the first lines of host defense. Its unique structure and diverse cell composition drive the host defense against the dissemination of pathogens through the blood^20, 21^. Kupffer cells (KCs) are the largest population of resident macrophages in the body and their primary function is to protect the liver from bacterial infections. Their location within the sinusoidal vascular space, predominantly in the periportal area, places these cells in the perfect position to clear gut-derived bacteria, endotoxins, debris, and metabolic waste arriving at the liver *via* the portal vein^22, 23^. KCs display high phagocytic and lysosomal activity, which highlights their specialization in monitoring and filtering the blood entering the sinusoids.

Coupling between EPO driven erythropoiesis, iron metabolism, and clearance of senescent and damaged erythrocytes by macrophages, is a key factor in red blood cell homeostasis^1^. KCs play a crucial role in hepatic iron metabolism and erythrocyte turnover^24, 25^. We and others have shown that macrophages from the spleen, peritoneum^26^ and BM^27–29^ express functional EPO-Rs and they respond to treatment with EPO. Nevertheless, an answer to the question of whether KCs are targets of EPO activity has remained elusive. Here we demonstrate that KCs express functional EPO-Rs and that EPO treatment promotes their proliferation and phagocytosis capability. Moreover, EPO stimulates KC-mediated attraction of CCR2^+^Ly6C^hi^ monocytes to the challenged liver *via* the production of their chemoattractant - CCL2.

## Results

### The RKC-2 Kupffer cell line expresses a functional EPO-R

**Figure 1:**
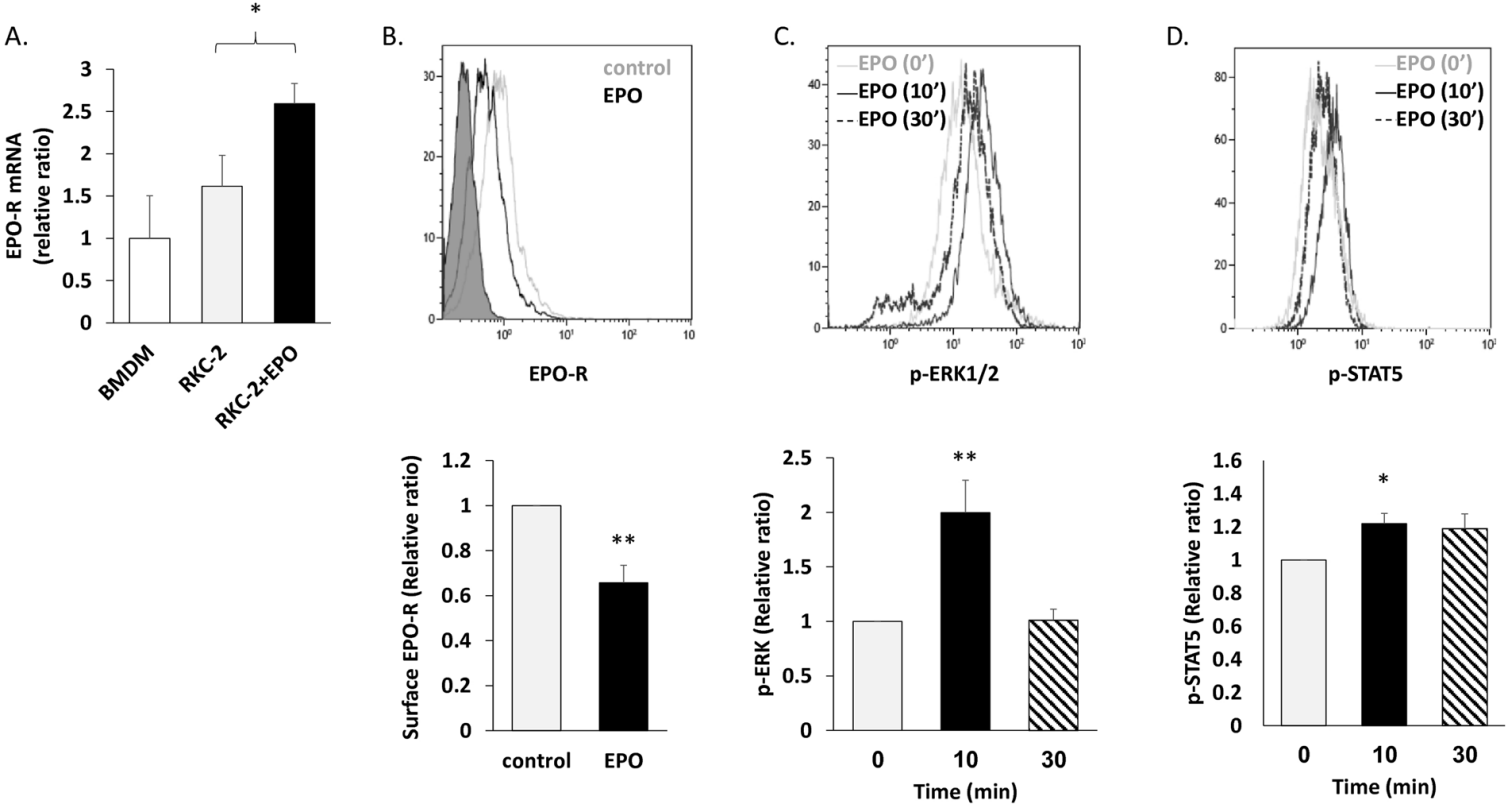
EPO regulates EPO-R expression and elicits downstream signalling in RKC-2 cells. All graphs represent mean ± SEM. (**A-B)** RKC-2 cells were cultured in the presence or absence of 5 U/ml EPO for 24 h. (**A**) EPO-R transcript levels were evaluated by RT-PCR, N = 5–8, *p < 0.05. Rat BMDM (positive control) were considered as 1. (**B**) EPO-R surface expression was evaluated by flow cytometry. Top: Grey and black line histograms depict surface EPO-R in control (considered as 1) and EPO-treated cells, respectively. Full histogram depicts FITC conjugated Goat anti mouse antibody. Bottom: Quantification of surface EPO-R, N = 7, **p < 0.01. (**C-D**) RKC-2 cells were stimulated for 0, 10 and 30 min with 10 U/ml EPO. Top: Grey, black and dashed histograms represent 0, 10 and 30 min EPO-treated cells, respectively. Bottom: The graphs depict quantification of mean fluorescence intensity (MFI) of flow cytometry analysis for p-ERK1/2 and p-STAT5, (**C**) and (**D**), respectively. Levels at 0 min were considered as 1, N = 4–5, **p < 0.01, *p < 0.05.

To address the question of whether KCs respond to EPO, we initially utilized the rat Kupffer cell line, RKC-2, as a model system^30^. We measured the expression levels of EPO-R transcripts and protein in RKC-2 cells in the presence or absence of EPO. Bone marrow-derived macrophages (BMDM) were referenced as a positive control for EPO-R expression^27, 29^. RT-PCR analysis detected EPO-R mRNA transcripts in these cells (Fig. 1A) and 24 h treatment with EPO led to a 60% increase (p < 0.05) in the levels of EPO-R transcripts. Flow cytometry analysis using a recently validated new monoclonal antibody directed against EPO-R^31^, further confirmed its expression at the protein level and a 24 h treatment with EPO led to a 34% decrease (p < 0.01) in the levels of cell surface EPO-R (Fig. 1B). These data are in accordance with previous reports demonstrating EPO mediated EPO-R endocytosis and internalization in various cell types^32–34^. In response to EPO binding, JAK2 is activated and phosphorylates Tyr residues on the EPO-R, which can then recruit and activate ERK1/2 and STAT5 among other secondary signalling molecules^35, 36^. ﻿In this regard,﻿ flow cytometry analysis demonstrated that EPO induces phosphorylation of ERK1/2 (Fig. 1C) and STAT5 (Fig. 1D), and that the response peaks at 10 minutes.

### EPO up-regulates phagocytosis by KCs

**Figure 2:**
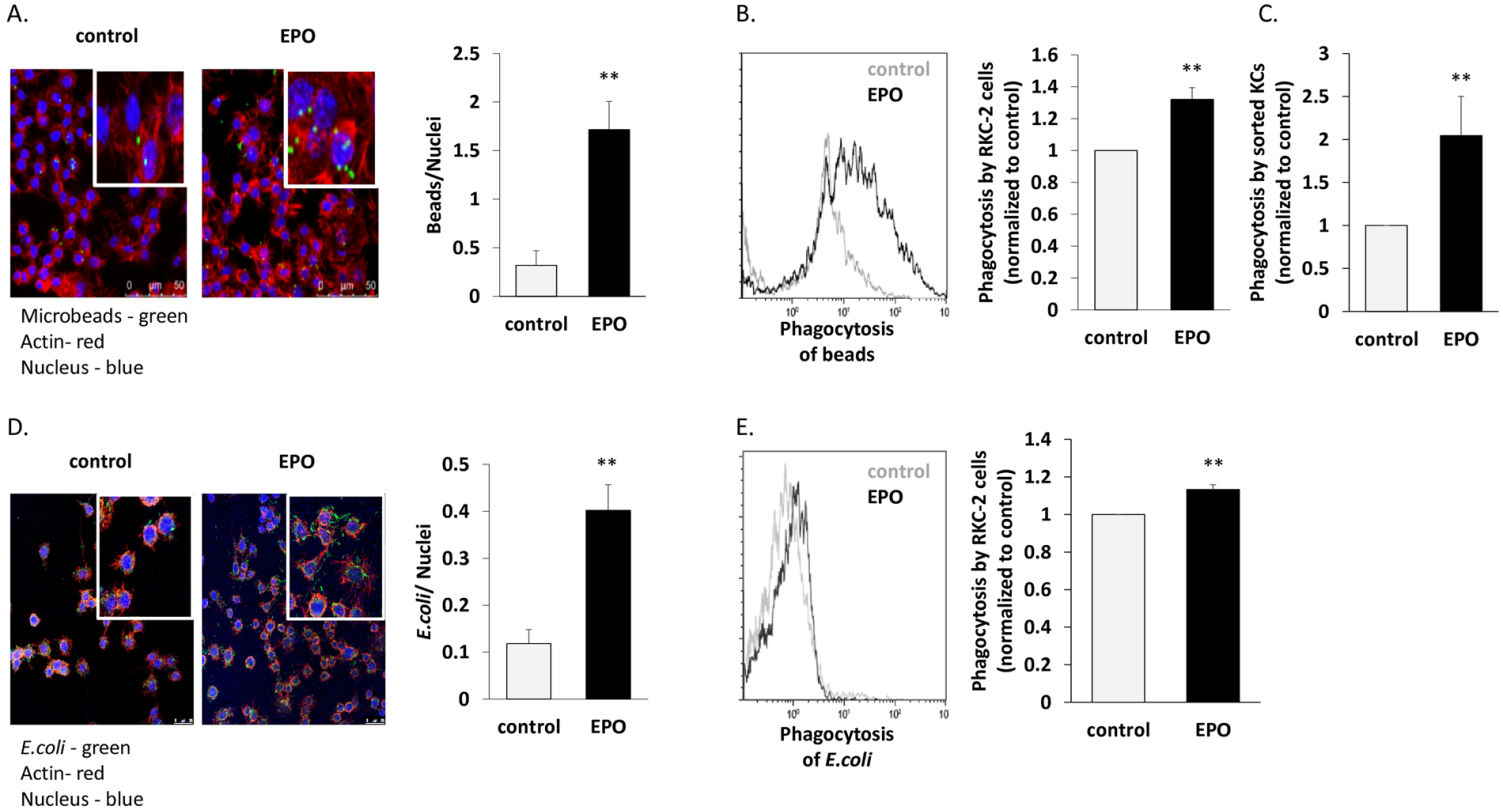
EPO increases phagocytosis by KCs. (**A**) 100,000 RKC-2 cells were cultured for 24 h. On the next day, DAPI-labeled RKC-2 cells were incubated ±10 U/ml EPO for 30 min, followed by 30 min incubation with fluorescently labeled polystyrene microbeads (green). Rhodamine phalloidin (red) and DAPI (blue) label actin and nuclei, respectively. The graph represents quantification of phagocytosed beads (mean ± SEM), N = 6, **p < 0.01. Bar = 50 µm. (**B**) Cells were treated ± EPO as in (**A**) and analyzed by flow cytometry for uptake of fluorescent beads (representative histogram). Grey and black represent control and EPO-treated cells, respectively. The graph depicts the fold increase in MFI reflecting the phagocytosis by cells treated with EPO compared to saline-treated cells (control, considered as 1). Results are mean ± SEM, N = 5, **p < 0.01. (**C**) 100,000 sorted KCs (CD45^+^/F4/80^hi^/CD11b^lo^) were treated as described in (**A**). Phagocytosis by EPO-treated cells was normalized to control (considered as 1). Results are mean ± SEM, N = 5, **p < 0.01. (**D**) 2.5 × 10^6^ RKC-2 cells were cultured for 24 h. On the next day, DAPI-labeled RKC-2 cells were incubated ±10U/ml EPO for 30 min followed by 1 h incubation with 4 × 10^7^ CFSE-labeled *E. coli* (green). Rhodamine phalloidin (red) and DAPI (blue) label actin and nuclei, respectively. The graph represents a quantification of phagocytosed CFSE labeled *E. coli* (mean ± SEM), N = 5, **p < 0.01, Bar = 25 µm. (**E**) Cells were treated ± EPO as in (**D**) and analyzed by flow cytometry to assay the uptake of CFSE-labeled *E. coli* (representative histogram). Grey and black represent control and EPO-treated cells, respectively. The graph depicts the MFI fold increase reflecting the phagocytosis of cells treated with EPO compared to saline-treated cells (control, considered as 1). Results are mean ± SEM, N = 6, **p < 0.01.

EPO enhances phagocytosis by peritoneal macrophages and BMDM *via* EPO-R^27, 29, 37, 38^. We thus questioned whether EPO could also increase the phagocytic capacity of KCs. We measured the effect of EPO on the ability of RKC-2 cells and sorted primary mouse KCs to phagocytose fluorescence-labeled polystyrene microbeads or CFSE (Carboxyfluorescein succinimidyl ester fluorescent cell staining dye) - labeled heat killed *E. coli*. The degree of phagocytosis of fluorescence-labeled polystyrene microbeads was determined by fluorescence microscopy (Fig. 2A) and flow cytometry analysis (Fig. 2B). Pre-treatment with EPO increased the capacity of RKC-2 phagocytosis of microbeads by 540% (p < 0.01) and 32% (p < 0.01), as determined by quantitative analysis of the fluorescent fields and flow cytometry, respectively. This was further confirmed in sorted KCs, where EPO increased the phagocytosis of microbeads by 2-fold (p < 0.01) (Fig. 2C). Similarly, EPO augmented RKC-2 cell phagocytosis of CFSE-labeled heat killed *E. coli*, as detected by fluorescence microscopy (Fig. 2D) and flow cytometry (Fig. 2E), by 340% increase (p < 0.01) and 13% increase (p < 0.01), respectively. Our results thus indicate that KCs are direct targets of EPO and that treatment with EPO enhances their phagocytic ability.

### EPO increases liver KC number and liver EPO-R gene expression

**Figure 3:**
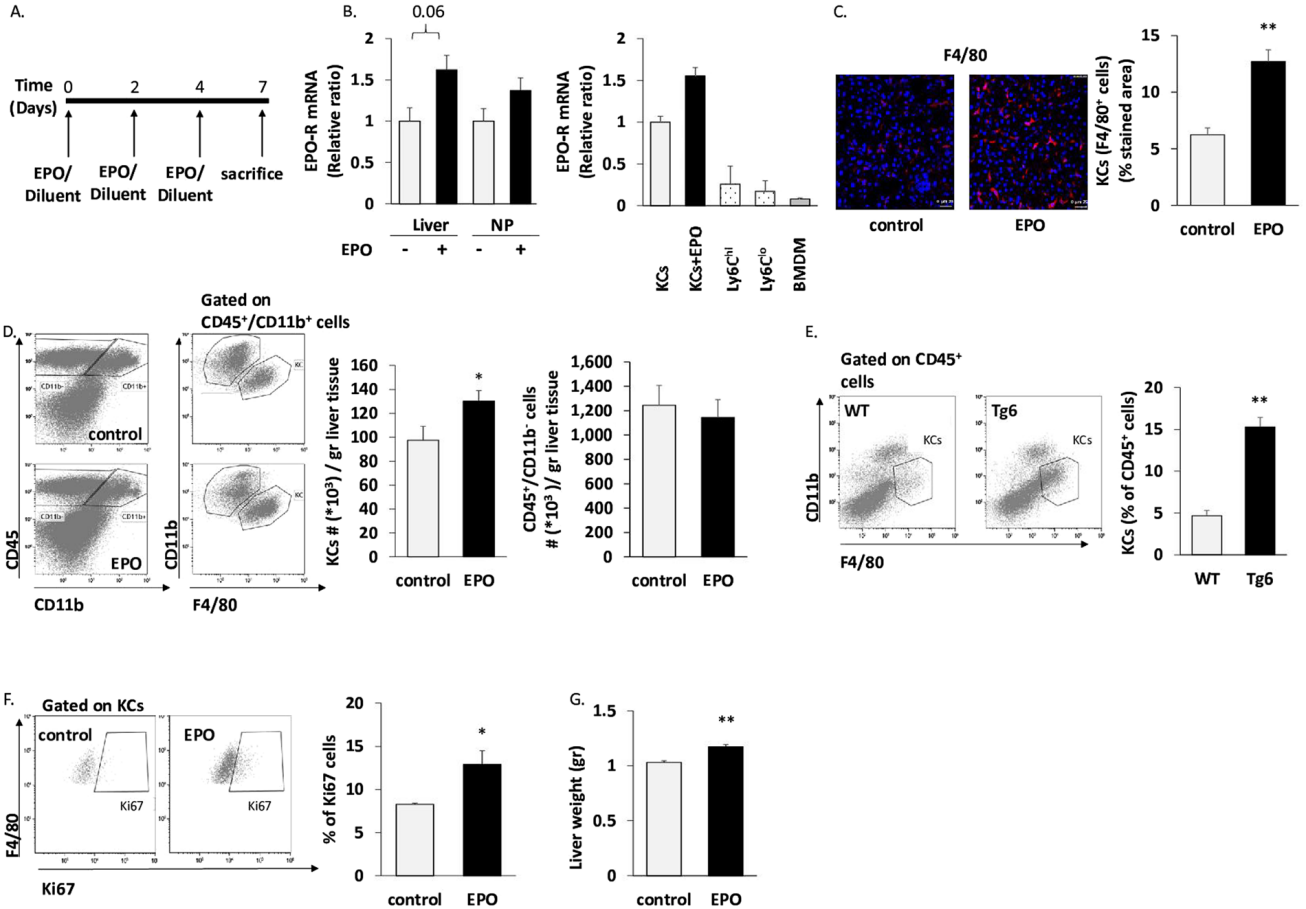
EPO-treatment increases KC number *in vivo*. Livers were harvested from C57BL/6 J mice injected 3 times on alternating days with 180 U EPO or diluent (control). (**A**) Schematic outline of EPO/diluent injections. (**B**) Left: EPO-R transcript levels in total liver (Liver) and in non-parenchymal cells (NP) from diluent and EPO-injected mice. Right: EPO-R transcript levels in sorted KCs (defined as CD45^+^/CD11b^int^/F4/80^hi^/CD64^+^ cells^39^) from diluent (KCs) or EPO-injected mice (KCs + EPO), were evaluated by RT-PCR. Graph represents EPO-R transcript levels ± SEM. BMDM served as a positive control^27^. Ly6C^hi^ = Ly6C^hi^ monocytes, Ly6C^lo^ = Ly6C^lo^ monocytes. (**C**) Frozen sections of the livers were stained with a purified antibody directed against F4/80 surface marker followed by Cy3 labeled Goat anti-Rat antibody, and viewed on confocal microscopy. Left: Representative analysis of livers from diluent (control) and EPO-injected mice. Right: Bars represent mean ± SEM, N = 5–6, **p < 0.01. Bar = 25 µm. (**D**) Liver non-parenchymal cells were analyzed by flow cytometry for CD45, F4/80 and CD11b. Left: Representative analysis of livers from diluent (control) and EPO-injected mice. Right: KCs or CD45^+^/CD11b^−^ (lymphocytes) cell number per gram liver tissue. Graphs represent mean ± SEM. For KCs: N = 12-13, *p < 0.05. For CD45^+^/CD11b^−^ cells: N = 7–8. (**E**) Livers of EPO-overexpressing mice (*Tg6*) or their WT littermates were harvested. Liver non-parenchymal cells were analyzed by flow cytometry for CD45, F4/80 and CD11b. Left: Representative analysis of livers from *Tg6* versus WT. Right: Graphs represent mean ± SEM, N = 6, **p < 0.01 (**F**) Left: Flow cytometry analysis of Ki67 labelling of gated KCs (representative plot). Right: quantification of Ki67 positive KCs, N = 4, *p < 0.05. (**G**) Graph represents liver weight as mean ± SEM, N = 7–9, **p < 0.01.

We next investigated *in vivo*, the effect of EPO on liver KCs. Livers from C75BL/6 J mice injected with diluent or EPO (3 injections of 180 U EPO on alternating days) were harvested (Fig. 3A). There was an EPO-associated increase in the levels of EPO-R mRNA in total liver and a tendency to increased EPO-R mRNA levels in the non-parenchymal cell fraction (Fig. 3B). Among these non-parenchymal cells, sorted KCs from EPO-injected mice tended to have higher EPO-R gene expression than their counterparts from diluent-injected mice (Fig. 3B). This EPO-associated increase *in vivo* was in line with our findings in the RKC-2 cells (Fig. 1A). Notably, EPO-R expression in Ly6C^hi^ and Ly6C^lo^ monocytes (CD45^+^/CD11b^hi^/F4/80^−/lo^/CD64^lo^/Ly6C^hi^ or Ly6C^lo^, respectively), which were sorted from untreated livers, as well as in BMDM (positive control), was profoundly lower than in KCs (Fig. 3B).

Immunofluorescence staining for the KC-associated macrophage marker F4/80 revealed a 2-fold (p < 0.01) increase in the number of KCs following EPO treatment (Fig. 3C). Flow cytometry analysis further confirmed a 33% increase (p < 0.05) in KC number normalized for tissue mass in the livers of the EPO-treated mice compared to diluent-treated mice (Fig. 3D). Of note, the numbers of hepatic lymphocytes, defined as CD45^+^/CD11b^−^ cells, were not affected by EPO administration (Fig. 3D). Similar EPO-governed effects were also observed in *Tg6* mice that are genetically modified to constitutively overexpress EPO in an oxygen-independent manner^40, 41^. Accordingly, we observed an approximately 3-fold increase (p < 0.01) in KC levels in *Tg6* mice compared to their wild type littermates (Fig. 3E). KCs are normally considered a self-sustaining population that is independent of adult BM hematopoiesis^39, 42–44^. To examine whether the EPO-induced increase in KCs results from local cell proliferation, we analyzed their expression of the Ki67 marker of active proliferation as previously reported^39^. Flow cytometry revealed a 56% increase (p < 0.05) in Ki67^+^ KCs following EPO administration (Fig. 3F), supporting the premise that EPO increases proliferation of KCs *in vivo*. These findings are in line with the EPO-associated increase in liver weight measured in EPO-treated mice compared to diluent-treated mice (10% increase, p < 0.01) (Fig. 3G).

**Figure 4:**
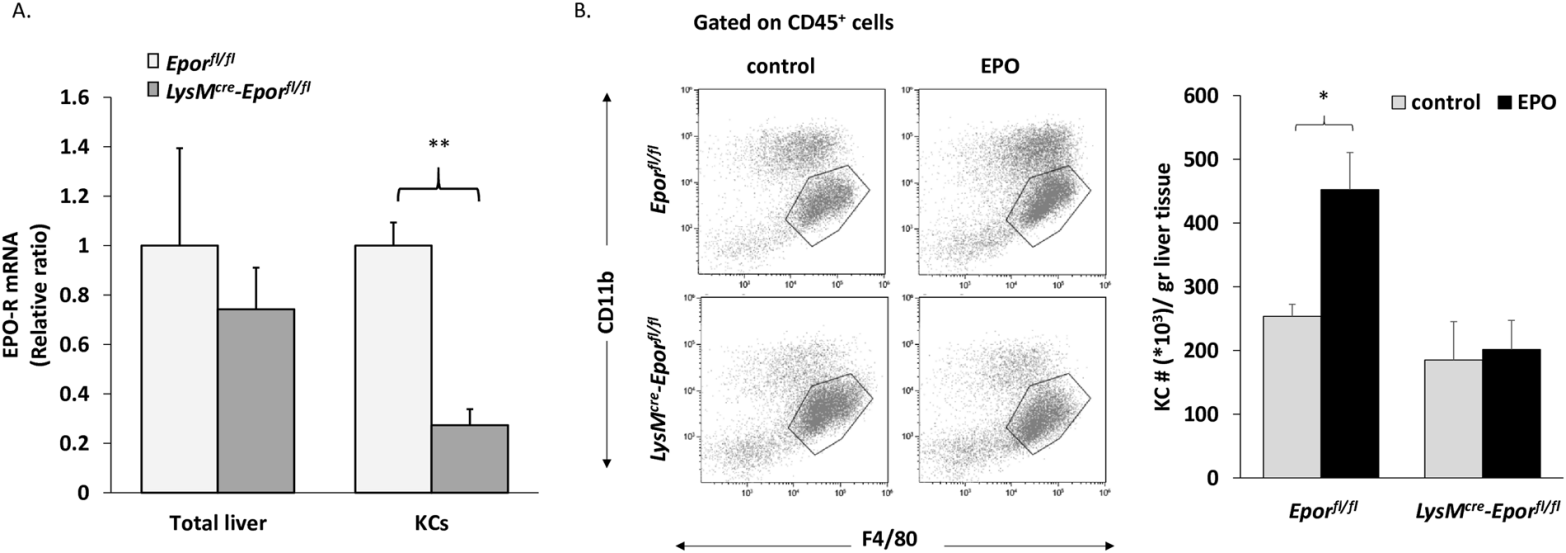
EPO effect on KC levels is abrogated in *LysM* ^*cre*^ *-Epor* ^*fl/fl*^. *Epor* ^*fl/fl*^ or *LysM* ^*cre*^ *-Epor* ^*fl/fl*^ mice were injected 6 times on alternating days over 2 weeks, with 180 U EPO or diluent (control). (**A**) EPO-R transcript levels in total liver cells and in sorted KCs (CD45^+^/F4/80^hi^/CD11b^lo^) from *Epor* ^*fl/fl*^ mice or *LysM* ^*cre*^ *-Epor* ^*fl/fl*^ mice were evaluated by RT-PCR. Graph represents EPO-R transcript levels ± SEM, N = 4–6, **p < 0.01. (**B**) Left: Representative analysis of livers from control and EPO-injected *Epor* ^*fl/fl*^ or *LysM* ^*cre*^ *-Epor* ^*fl/fl*^ mice. Right: KC number per liver weight (gr). Graphs represent mean ± SEM, N = 4–9, *p < 0.05.

We next sought to study whether the EPO-mediated increase in KC number is driven by a direct intrinsic cellular mechanism in these cells. An EPO-R-deficiency in the myeloid lineage was obtained by generating *LysM* ^*cre*^ *-Epor* ^*fl/fl*^ mice. In the liver of these mice, Cre expression driven by the M lysozyme (LysM) promoter mainly affects KCs^45^, though other myeloid cells including macrophages in other tissues are also targeted. While total liver cells from *LysM* ^*cre*^ *-Epor* ^*fl/fl*^ mice did not exhibit a significant change in *Epor* gene expression levels compared to total liver cells from the relevant controls (*Epor* ^*fl/fl*^ mice), KCs sorted from these mice displayed a 73% decrease (p < 0.01) in *Epor* transcript levels (Fig. 4A). EPO significantly increased the number of KCs in the control *Epor* ^*fl/fl*^ mice, ﻿but﻿ had no effect on KC numbers in the *LysM* ^*cre*^ *-Epor* ^*fl/fl*^ mice (Fig. 4B). These results suggest that direct EPO signalling can instruct KC proliferation. However, we cannot exclude the possibility that EPO signalling in other myeloid cells may also contribute to the increased proliferation of KCs.

### EPO increases CCL2-mediated recruitment of Ly6C^hi^ monocytes to the injured liver

**Figure 5:**
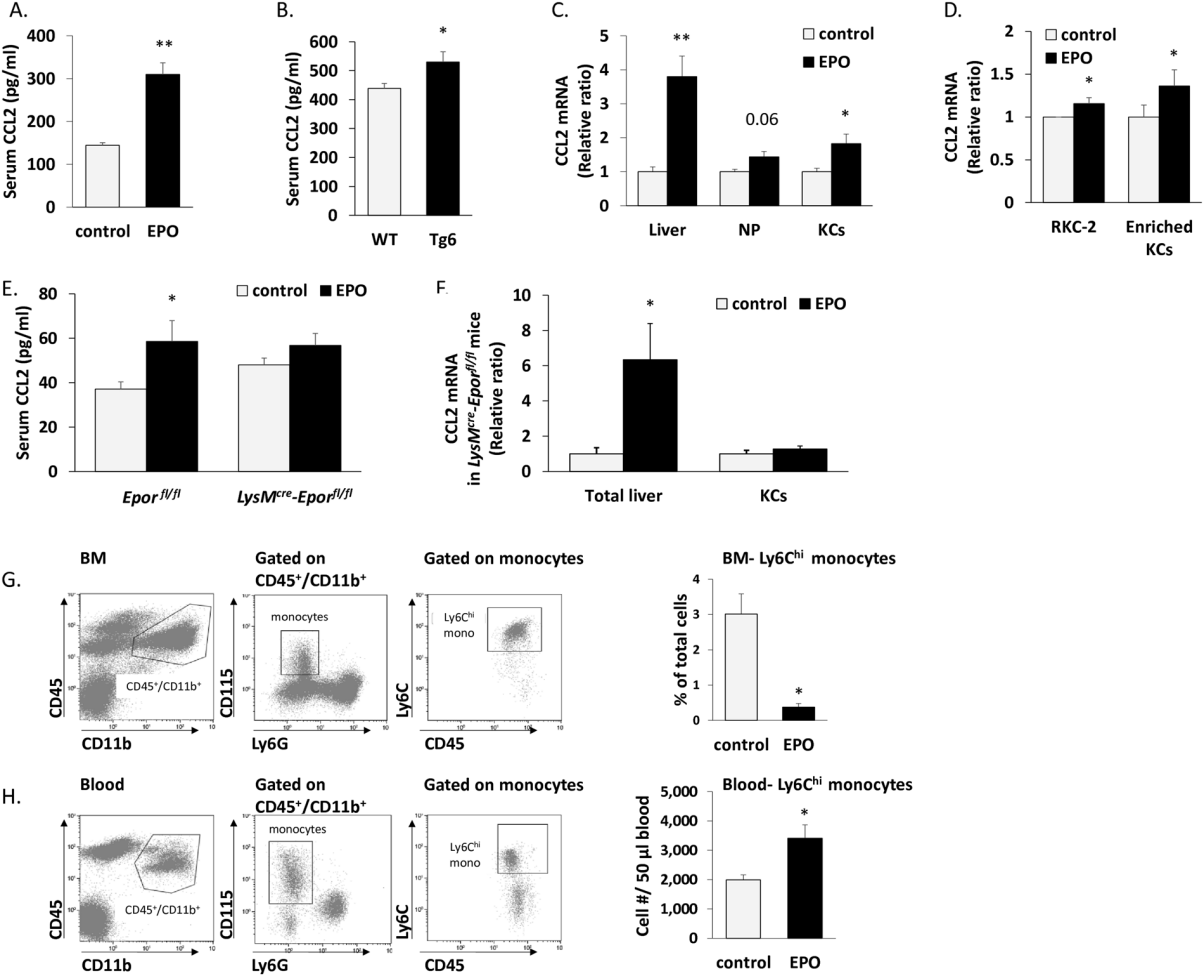
EPO increases CCL2-deriven egress of Ly6C^hi^ monocytes from BM. (**A**) Serum CCL2 levels in diluent (control) or EPO-injected mice detected by ELISA, graphs represent mean ± SEM, N = 5, **p < 0.01. (**B**) Serum CCL2 levels of EPO-overexpressing mice (*Tg6*) or their WT littermates detected by ELISA, graphs represent mean ± SEM, N = 7–9, *p < 0.05. (**C**) CCL2 transcript levels in total liver cells (Liver), non-parenchymal cells (NP) and KCs from diluent (control) or EPO-injected mice, were evaluated by RT-PCR, graphs represent mean ± SEM, N = 4–7, **p < 0.01, *p < 0.05. (**D**) CCL2 transcript levels of RKC-2 cells treated with 5 U/ml EPO or diluent (control) *in vitro* (N = 4) or enriched KCs treated with EPO or diluent (control) *in vitro* (N = 4) were evaluated by RT-PCR, graphs represent mean ± SEM, *p < 0.05. (**E**) Serum CCL2 levels of diluent or EPO-injected *Epor* ^*fl/fl*^mice or *LysM* ^*cre*^ *-Epor* ^*fl/fl*^ mice detected by ELISA, graphs represent mean ± SEM, N = 7–13, *p ≤ 0.05. (**F**) CCL2 transcript levels in total liver cells and in sorted KCs (CD45^+^/F4/80^hi^/CD11b^lo^) from *LysM* ^*cre*^ *-Epor* ^*fl/fl*^ mice were evaluated by RT-PCR. Graph represents CCL2 transcript levels ± SEM, N = 4–6, *p < 0.05. (**G**) BM were analyzed for CD45, CD11b, Ly6G, CD115 and Ly6C by flow cytometry. Left: Gating strategy for the definition of monocytes in BM. Right: bar graphs indicate Ly6C^hi^ monocytes as a percentage of total BM cells from diluent (control) or EPO-injected mice, N = 7–13, *p < 0.05. (**H**) Peripheral blood was analyzed for CD45, CD11b, Ly6G, CD115 and Ly6C by flow cytometry. Left: Gating strategy for the definition of monocytes in blood. Right: bar graphs indicate the number of Ly6C^hi^ monocytes per 50 µl blood from diluent (control) or EPO-injected mice, N = 11–12, *p < 0.05.

Following liver injury, liver resident cells including KCs initiate an innate immune response by releasing chemokines such as CCL2, which attracts Ly6C^hi^ monocytes^39, 46–48^. Specifically following challenge with stressed erythrocytes, KCs up-regulate CCL2 expression^25^. EPO treatment induced a 1.5-fold increase (p < 0.01) in serum CCL2 (Fig. 5A), which was also significantly higher (p < 0.05) in constitutively EPO-overexpressing *Tg6* mice compared to their wild type counterparts (Fig. 5B). Cells extracted from total livers of  EPO treated mice displayed a 3.7-fold-increase (p < 0.01) in the levels of CCL2 transcripts (Fig. 5C). A breakdown analysis of CCL2 transcripts in the livers of EPO-injected mice revealed a 1.43-fold-increase (p = 0.06) in non-parenchymal cells, and a 1.82-fold increase (p < 0.05) in sorted KCs, compared to their control counterparts (Fig. 5C). Moreover, both RKC-2 cells and enriched KC cultures (95% positive for F4/80, *see* Fig. S1) treated with EPO *in vitro*, showed a significant elevation of CCL2 transcripts compared to diluent-treated cells (Fig. 5D). Importantly, while *LysM* ^*cre*^ *-Epor* ^*fl/fl*^ mice did not display an EPO-driven increase in the levels of serum CCL2 (Fig. 5E) and CCL2 transcripts in sorted KCs (Fig. 5F), they still exhibited an increase in the levels of CCL2 transcripts in total liver cells (Fig. 5F). Thus, EPO may induce C﻿CL2 production by other EPO-R-expressing liver cells, such as hepatocytes. Collectively, these findings strongly endorse a central role for EPO in the promotion of KC-governed CCL2 production.

Egress of Ly6C^hi^ monocytes from the BM to the circulation is known to be mediated by CCR2^49^. As expected from its positive effect on CCL2 expression, EPO administration led to an 8-fold (p < 0.05) decrease in the levels of CD45^+^CD11b^+^CSF1R(CD115)^+^/Ly6C^hi^ monocytes in the BM (Fig. 5G), while the numbers of their mature descendants in the blood were increased by 1.7-fold (p < 0.05) (Fig. 5H). Interestingly, EPO treatment had no effect on the expression of other chemokines such as CCL5, CCL8, and CXCL2, although there was a marked reduction in the fractalkine chemokine CX_3_CL1, and an increase in the expression of the neutrophil chemoattractant CXCL1 (Fig. S2). Furthermore, livers from EPO-treated mice exhibited elevated expression of the genes encoding for EPO and the anti-inflammatory cytokines IL-10 and TGFβ, with unchanged expression of the pro-inflammatory mediators IFNγ and nitric oxide synthase 2 (NOS2) (Fig. S2). To study whether these genes are expressed by KCs, we revisited our gene expression database of sorted KCs from steady state livers^39^. To establish the background expression level, their gene expression was compared with that of adiponectin (*Adipoq*), which is uniquely expressed by adipocytes. The gene expression of C-type lectin domain family 4 member F (*Clec4f*), previously defined as a KC specific marker^44^, was used as a positive control. KCs exhibited clear expression of the genes encoding for IL-10, TGFβ1, CCL2, and CXCL1, as well as EPO-R, while they were negative for EPO and CX_3_CL1 (Fig. S2).

**Figure 6:**
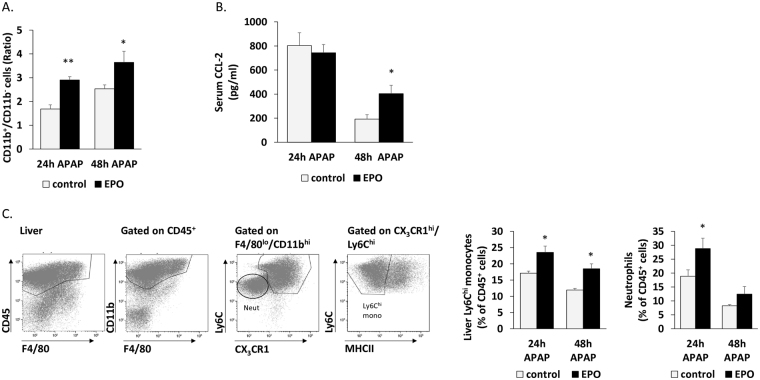
EPO increases recruitment of Ly6C^hi^ monocytes and neutrophils during AILI. (**A**) Liver non-parenchymal cells were analyzed by multi-parameter flow cytometry. The gating strategy for the definition of CD11b^+^ (myeloid cells) or CD11b^−^ (lymphocytes) in the APAP-challenged livers was defined by using CD45, F4/80 and CD11b. Bar graphs indicate CD11b^+^/ CD11b^−^ cell ratio from diluent (control) or EPO-injected mice, 24 h or 48 h post injection with APAP, N = 5, **p < 0.01, *p < 0.05. (**B**) Serum CCL2 levels detected by ELISA in diluent (control) or EPO-injected mice, 24 h or 48 h after treatment with APAP, N = 4–10, *p ≤ 0.05. (**C**) Liver non-parenchymal cells were analyzed by flow cytometry. The gating strategy for the definition of Ly6C^hi^ monocytes or neutrophils in the APAP-challenged liver was defined by using CD45, F4/80, CD11b, Ly6C, CX_3_CR1 and MHCII. Bar graphs indicate Ly6C^hi^ monocytes or neutrophils as a percent of CD45^+^ liver cells from diluent (control) or EPO-injected mice, 24 h or 48 h post injection with APAP, N = 4–10, *p < 0.05. All graphs represent mean ± SEM.

We next examined the effect of EPO on the active recruitment of monocytes to the liver in a well-characterized murine model of AILI, induced by an overdose of N-acetyl-p-aminophenol (APAP)^39^. In this model, Ly6C^hi^ monocytes massively infiltrate the injured liver during the necro-inflammatory phase (first 24 h) to become the dominant mononuclear phagocyte subset in the tissue^39^. Mice were treated with EPO or saline 24 h before APAP administration and then together with the APAP treatment. The impact of EPO treatment on liver infiltration of Ly6C^hi^ monocytes was analyzed 24 h after APAP challenge (24 h APAP), as this is the time at which they reach maximum accumulation^39^. Livers were also evaluated 48 h following APAP challenge, when Ly6C^hi^ monocytes are still present in the liver and differentiation towards Ly6C^lo^ macrophages is not yet complete^39^. In this “48 h APAP” group, an additional EPO or saline treatment was administrated 24 h after the APAP injection. CD11b^+^/CD11b^−^ cell ratio basically indicates the distribution of phagocytes and lymphoid cells. Treatment with EPO led to a 72% and 44% increase (p < 0.01 and p < 0.05, respectively) in phagocyte/lymphoid cell ratio in the liver, at 24 and 48 h following APAP injection, respectively (Fig. 6A). These results are coupled with the lack of EPO effect on the lymphocyte population (Fig. 3D). Treatment with EPO led to a 37% increase (p < 0.05) and 54% increase (p < 0.05) in Ly6C^hi^ monocyte levels (defined as CD45^+^CD11b^+^Ly6C^hi^F4/80^lo^CD64^lo^CX_3_CR1^+^MHCII^lo^) in the liver, at 24 and 48 h after APAP injection, respectively (Fig. 6C). Serum levels of CCL2 were similarly high at 24 h post APAP, which may be expected given that this is the time of peak induction following AILI^39^. Notably, EPO treatment maintained higher levels of CCL2 [2-fold increase] in the serum at 48 h, when it was already reduced in the control group (Fig. 6B).

We have recently demonstrated a spatial and temporal overlap in the migration pattern of liver infiltrating Ly6C^hi^ monocytes and neutrophils following AILI^50^. Given the increased expression of the neutrophil chemoattractant CXCL1 in EPO treated livers (Fig. S2), and the observation that KCs express this chemokine (Fig. S2), we next followed the effect of EPO treatment on neutrophil recruitment to the injured liver. Indeed, flow cytometry analysis revealed a significantly higher infiltration of neutrophils (defined as CD45^+^CD11b^+^Ly6C^lo^F4/80^-^CD64^-^CX_3_CR1^-^) at 24 h following AILI (Fig. 6C). Moreover, KCs exhibited increased production of CXCL1 transcripts in response to direct EPO treatment (Fig. S2), suggesting that KC-EPO axis  may be involved in the attraction of neutrophils to the injured liver. Nevertheless, CXCL1 gene expression levels were significantly up-regulated in EPO-treated *LysM* ^*cre*^ *-Epor* ^*fl/fl*^ mice (Fig. S2), suggesting that EPO-governed signalling in myeloid cells, and in particular KCs, is not solely responsible for the elevated expression of this neutrophil chemoattractant. Collectively, these results support a role for EPO in promoting KC-mediated recruitment of Ly6C^hi^ monocytes and neutrophils in response to liver injury.

## Discussion

While the main classical role of EPO is considered to be the regulation of red blood cell production, non-erythroid effects of EPO have been repeatedly reported^7–15^. These findings underline the importance of investigating the global actions of rHuEPO and its derivatives in pre-clinical and clinical settings. The goal is to maximize benefits, while reducing harm. One of the non-erythroid targets of EPO is the immune system. Work with mouse models and clinical studies^51–53^, including our own^27, 54–59^, have concluded that EPO treatment modulates immunological functions. As an example, EPO enhanced BMDM phagocytic activity and nitric oxide secretion in mice, and led to augmented IL-12 secretion and decreased IL-10 secretion in response to lipopolysaccharide^27^. Moreover, dendritic cells derived from rat BM responded to EPO stimulation with increased expression of dendritic cell activation markers and a higher allostimulatory capacity for T cells^51^. In chronic renal failure patients, who did not require hemodialysis, administration of rHuEPO stimulated rejuvenation of cellular CD8^+^ T-dependent immunity^53^. Importantly, we^29, 54–56, 58, 60^ and others^61, 62^ have linked EPO administration to prolonged survival and improved immunological functions in multiple myeloma (MM) mouse models and MM patients. Moreover, EPO treatment in hematologic patients improved the response to influenza vaccination^63^ and normalized the levels of impaired CD4^+^ and CD8^+^ T cell and CD4^+^CD25^+^ cell numbers in low risk myelodysplastic syndrome patients (MDS)^64^.

KCs, the resident tissue macrophages of the liver, play a central role in the pathogenesis and resolution of various liver diseases^65^. We^27, 57, 59^ and others^26^ have found functional EPO-R on macrophages derived from BM, peritoneum and spleen. By data mining in a gene expression database of steady state KCs^39^, we show here that these cells express EPO-R at the gene level. This has previously been shown for rat KCs^66^. Nevertheless, it was not yet clear whether this receptor is functional and the nature of the effect of EPO on KC immunity remained elusive. Here we demonstrate for the first time that KCs express a functional EPO-R, which activates downstream signalling in response to EPO, to regulate KC number and function. We also show that EPO promotes KC-mediated Ly6C^hi^ monocyte and neutrophil recruitment to the challenged liver *via* production of CCL2 and CXCL1, respectively.

Being strategically positioned in the liver, KCs are characteristically involved in the phagocytosis of debris, pathogens, and apoptotic, damaged or senescent cells (including erythrocytes)^67–69^. They display high phagocytic and lysosomal activities, which relate to their specialization in filtering blood that enters the sinusoids. Thus, they are considered to play a key role in immune surveillance, iron metabolism, and detoxification in the liver. Our results demonstrate that treatment with EPO elicits EPO-R downstream signalling in cultured KCs and increases the capacity of cultured and sorted primary KCs to phagocytose. Therefore, EPO may directly license the ability of KCs to reduce any excess of red blood cells in the liver after its administration. This is also in line with a previous report indicating that peritoneal macrophages from EPO-treated mice, display enhanced apoptotic cell clearance through the peroxisome proliferator activated receptor-γ (PPARγ)^26, 37, 38^ as well as increased removal of debris^37^.

Under homeostatic conditions, KCs are replenished by a self-renewal process that is independent of adult BM hematopoiesis^39, 42–44^. Our results indicate that the increase in KCs following EPO administration *in vivo* is mediated, at least in part, by their local proliferation. Emerging evidence indicates that macrophage niche availability restricts tissue resident macrophage self-maintenance capacity^70^. The expansion in liver tissue mass following EPO treatment that we report here, may be accompanied by the inauguration of new macrophage niches that are then available for the arrival of *de-novo* KCs generated by self-proliferation of the already existing resident KC population. This is supported by our observation that the EPO-driven increase in KC levels is abrogated in *LysM* ^*cre*^ *Epor* ^*fl/fl*^ mice, where EPO-R-deficiency is targeted to myeloid cells. KCs are the main myeloid resident cell population in the normal liver^45^, and they exhibit a 73% reduction in EPO-R expression in the *LysM* ^*cre*^ *Epor* ^*fl/fl*^ mice. These results thus suggest that EPO can directly instruct KC proliferation; although we cannot exclude an alternative possibility that EPO indirectly affects KC expansion. Under certain circumstances in which resident macrophage niches are open, circulating Ly6C^hi^ monocytes can also differentiate into self-maintaining tissue-resident macrophages that resemble their embryonic counterparts^70^. Indeed, in cases when the KC pool is diminished such as following lethal irradiation or diphtheria toxin induced ablation^44^, or more physiologically, following *Listeria monocytogenes-*induced KC necroptosis^71^, Ly6C^hi^ monocytes are capable of reconstituting the KC compartment. Our results, demonstrating that EPO mediates the egress of Ly6C^hi^ monocytes from the BM to the circulation, suggest that these cells may also contribute to the expansion of KC population.

Prolonged EPO administration, as is often prescribed in clinical conditions such as anemia, may pose a challenge to the liver. Our results suggest that in such a scenario, activation of the EPO/EPO-R axis in KCs induces their proliferation and phagocytic activity, and thus licenses them to clear damaged and stressed red blood cells. Further substantiating this argument, are the results showing that EPO stimulates CCL2 production in KCs, and promotes the egress of Ly6C^hi^ monocytes from the BM to the circulation. It has been recently shown in a mouse model of hemolytic anemia, that CCL2-dependent Ly6C^hi^ monocyte recruitment to the liver is important for the clearance of senescent and dying erythrocytes, and protection from iron toxicity^25^. Therefore, EPO-mediated up-regulation of CCL2 production by KCs may be an additional pathway participating in the clearance of excess erythrocytes in the liver.

The EPO mediated *in vivo* increase in serum and liver CCL2 (protein and transcripts, respectively), and the absence of this EPO-driven phenotype in KCs isolated from *LysM* ^*cre*^ *-Epor* ^*fl/fl*^ mice, strongly suggest that activation of EPO-R in KCs contributes to this EPO mediated response. It should be noted that in *LysM* ^*cre*^ mice, Cre-mediated deletion, specificity and efficiency is mainly restricted to macrophages, monocytes and neutrophils with little effect on other immune cells such as T cells, B cells, NK cells, eosinophils, dendritic cells, and mast cells^72^. Indeed, the depletion of EPO-R in the KC compartment, but not in total liver preparations from *LysM* ^*cre*^ *-Epor* ^*fl/fl*^ mice, indicates that EPO-R is mainly restricted to KCs. In addition, we show that monocytes have significantly less EPO-R than KCs and although we could not directly examine the expression of a functional EPO-R in sorted neutrophils due to their poor viability following isolation, a previous study has indicated that extended EPO administration has no effect on neutrophil pools *in vivo* ^73^. Moreover, neutrophils were profoundly outnumbered by KCs in the liver following EPO treatment (Data not shown). We provide a direct evidence for the existence of an EPO-KCs-CCL2 axis. First, EPO treatment stimulated CCL2 production in a Kupffer cell line as well as in enriched cultured primary KCs. Second; the EPO-mediated increase in CCL2 transcripts was abrogated in KCs sorted from *LysM* ^*cre*^ *-Epor* ^*fl/fl*^ mice and not in KCs from the control mice. Nevertheless, we cannot negate the participation of other cells in the liver that can produce monocyte and neutrophil attracting chemokines. This is especially true for hepatocytes, which are a prominent source for specific chemokines such as CCL2 under various challenging conditions^74^. As hepatocytes also express a functional EPO-R^66, 75^ they may contribute to the EPO driven increase in total liver CCL2 transcripts and serum CCL2. In support of this, we show that the EPO driven increase in CCL2 gene expression is maintained in the livers of *LysM* ^*cre*^ *-Epor* ^*fl/fl*^ mice, whose KCs lack EPO-R expression. Therefore, EPO may favor hepatocyte-mediated recruitment of Ly6C^hi^ monocytes to the challenged liver.

We show that EPO transcripts are elevated in the liver following systemic EPO treatment, although the mechanism of action and the nature of the cells that mediate this response remain elusive. Notably, extra-renal EPO production has been demonstrated in resident liver cells including hepatocytes and perisinusoidal Ito cells^44^, and was shown to increase following an acute-phase response in a rat model^46^. Local elevation in EPO levels has been documented in various liver pathologies, including AILI, and iron loading may perpetuate hepatic damage and compromise healing^76, 77^. Moreover, EPO has been suggested to play a protective role in the resolution of liver injury^16–18^. Hepatic infiltration of Ly6C^hi^ monocytes was reported in various acute or chronic liver injuries^39, 78–81^ and we have previously shown that Ly6C^hi^ monocytes in the liver give rise to pro-restorative ephemeral macrophages during AILI^39^. This is in accordance with our results here showing that the administration of EPO in an acute model of AILI maintains high levels of CCL2 in the serum and promotes the downstream recruitment of Ly6C^hi^ monocytes to the liver. Since KCs are also pivotal players in the recovery from AILI^82^, the role of EPO in liver resolution may therefore be through stimulation of KC proliferation, phagocytosis and recruitment of Ly6C^hi^ monocytes. Interestingly, neutrophil recruitment to the injured liver was also significantly augmented by EPO treatment at 24 h following APAP. The CXCL1 neutrophil chemoattractant was elevated in response to a week of EPO treatment in steady state mice. Notably, we show that KCs express elevated CXCL1 transcripts in response to EPO, raising the possibility that these cells also drive neutrophil mobilization in response to EPO. However, the observation that EPO also increased the levels of CXCL1 transcripts in the liver of *LysM* ^*cre*^ *-Epor* ^*fl/fl*^ mice, suggests that EPO-responsive cells other than KCs may produce CXCL1. Hepatocytes have been shown to express a functional EPO-R^66, 75^ and these cells may be candidates to mobilize neutrophils from the BM *via* their release of CXCL1^83^. In conclusion, we report a novel function of EPO associated with the regulation of KC number and function in the liver.

## Materials and Methods

### Mice

Female C57BL/6J-OlaHsd mice (8–12-weeks old) were purchased from Envigo Laboratories (Israel, formerly Harlan Laboratories). Mice were injected subcutaneously (s.c.) three times (every other day during 1 week) with 180 U rHuEPO (EPO, Epoetin α, Eprex®, Janssen), with diluent (saline) as previously described^27^, or as otherwise indicated. Mouse handling and the experimental procedures were approved by the Institutional Animal Care and Use Committee of the Tel-Aviv University (permit number: M-15-046), or Sourasky Medical Center Animal facility (5-6-16). All the experiments were conducted in accordance with the approved guidelines.

C57BL/6 J mice at the age of 9–11 weeks, as well as *Epor* ^*fl/fl*^ and *LysM* ^*cre*^ *-Epor* ^*fl/fl*^ mice, were injected subcutaneously (s.c.) six times (every other day for 2 weeks) with 180 U rHuEPO or with diluent (saline). Mouse handling and the experimental procedures were approved by the Institutional Animal Care and Use Committee of the Tel-Aviv University (permit number: M-14-093) in accordance with the approved guidelines.

### Rat RKC-2 - Cell line

The immortalized rat Kupffer cell line 2, RKC-2^30^ was kindly provided by Prof. T. Hunter (USF, University of South Florida). Cells were maintained in RPMI 1640 medium supplemented with 10% FCS at 37 °C and 5% CO_2_. For experiments, RKC-2 cells were cultured for 24 h or as otherwise indicated, in RPMI 1640, containing EPO as indicated below.

### Kupffer cells enrichment *ex vivo*

KCs were separated from hepatocytes and other sinusoidal cells by gradient centrifugation. Specifically, mice were perfused with 30 ml of cold PBS and the liver was excised. Livers were cut into small fragments, incubated with mixing (37 °C, 250 rpm for 45 min) with 5 ml digestion buffer [5% FCS, 0.5 mg/ml collagenase IV (Sigma-Aldrich, Rehovot, Israel, C5138-500MG) in PBS] and filtered through 200 μM wire mesh. Cells were suspended in 5 ml RPMI 1640 and centrifuged at 300 g for 5 min at 4 °C, then the top aqueous phase was discarded. The cell sediments were resuspended in 10 ml RPMI 1640 and centrifuged at 50 g for 3 min at 4 °C. The top aqueous phase (cleared cell suspension) was transferred into a new 10 ml centrifuge tube and centrifuged at 300 g for 5 min at 4 °C, then the top aqueous phase was discarded, and the cell sediments were reserved. The cell sediments mainly contained non-parenchymal cells of the liver including KCs, sinusoidal endothelial cells, and satellite cells. Selective adherence to plastic as described by Blomhoff *et al*.^84^ was used to purify this cell population further. The cells were then seeded into 10 cm plates in DMEM containing 10% FBS, 1% L-Glutamine (L-Glu) and 0.1% Penicillin Streptomycin (PS), and incubated for 1 h in a 5% CO_2_ atmosphere at 37 °C. Non-adherent cells were then removed from the dish by gently washing with PBS, to leave the adherent KCs. Cells were grown for 7–10 days in culture until they displayed morphologic features typical of macrophages.

### Erythropoietin (EPO)

GMP-manufactured sterile syringes containing rHuEPO (Epoetin alfa, Eprex®) as used for patient care, were kindly provided by Janssen Cilag, Israel, and employed throughout this study, thus ensuring the absence of toxins in the rHuEPO preparation.

### APAP administration

Mice were fasted overnight for 12 h prior to administration of diluent (saline) or 300 mg/kg APAP (CAT# 7085, SIGMA) by intraperitoneal injection (i.p). Drinking water was provided at the same time as APAP administration and access to food was restored 2 h later.

### Surface EPO-R expression

Rat RKC-2 cells (1 × 10^5^ cells) were incubated in 6-well plates and stimulated in the presence or absence of EPO 5 U/ml. After 24 h, the cells were collected and labeled with anti EPO-R antibody (GM1202, Aldevron, Freiburg^31^), followed by FITC conjugated Goat anti mouse antibody (Jackson ImmunoResearch, CAT#115-0095-003). The stained cells were examined by Gallios^TM^ Flow cytometer and analyzed using Kaluza Analysis Software (Beckman Coulter, Nyon Switzerland).

### PhosphoFlow of RKC-2 cell line

RKC-2 cells were starved for 1 h and aliquoted to Eppendorf tubes (10^6^ cells/ 90 µl RPMI 1640). They were then incubated (37 °C, 5 min) followed by activation with 10 U/ml EPO for 0, 10, and 30 min. The reaction was stopped with 10 µL of 37% formalin, giving a final concentration of 3.7% formalin. Cells were incubated for 1 min at 37 ^°^C, and then cooled on ice for 5 min and centrifuged (3,000 rpm for 2 min). After this, the cells were washed once with 1% BSA/PBS and centrifuged (3,000 rpm for 2 min). Staining for the intracellular proteins [(PE eFluor 610 pSTAT5 (eBioscience 61-9010-42) and p-ERK (sc-7383) followed by an FITC conjugated Goat anti-mouse antibody] was done in Saponin (Invitrogen) 1%BSA/PBS. The cells were incubated for 1 h in the dark at room temperature and then centrifuged and resuspended in Flow buffer. The stained cells were examined by Gallios^TM^ Flow cytometer and analyzed using Kaluza Analysis Software (Beckman Coulter, Nyon Switzerland).

### Macrophage phagocytosis of fluorescently labelled *E. coli* or fluorescently labelled beads

2 × 10^5^ RKC-2 cells were seeded in 60 mm plates overnight at 37 °C to allow cell adhesion. The next day, *E. coli* (strain BL21) were heat killed by incubation at 65 °C for 1 h and washed twice with PBS. They were then labelled with CFSE [M CFDA (Molecular Probes)] for 15 min at room temperature and washed thoroughly in PBS. RKC-2 cells were incubated ±10 U/ml EPO followed by stimulation with CFSE-labeled *E coli* (40 million cells) or fluorescently labeled beads diluted 1:800 (Fluoresbrite TM Plain YG 1.0 Micron Microspheres, CAT#17154, Polysciences Inc) for the indicated concentrations and time points. Free bacteria that had not been phagocytosed by RKC-2 cells were washed away. Cells were aliquoted to FACS tubes and analyzed on a Gallios^TM^ Flow Cytometer (Beckman Coulter). Similarly, 1 × 10^5^ sorted KCs (CD45^+^/F4/80^hi^/CD11b^lo^) were cultured in 6-well plates for 24 h. On the next day, the cells were incubated ±10 U/ml EPO for 30 min, followed by 30 min incubation with fluorescently labeled polystyrene microbeads. Cells were analyzed by flow cytometry for uptake of fluorescent beads.

### Immunofluorescence assay

RKC-2 cells (10^5^ cells in 500 µl per well in 24 well plates) were treated with EPO and *E. coli* or beads (as described above) then washed three times with PBS and fixed in 4% paraformaldehyde diluted in PBS for 30 min at room temperature. The cells were then washed three times with PBS, perforated by incubation in quenching buffer (0.1% Triton × 100, 2% BSA, 5% FBS in PBS) for 15 min at room temperature, and stained with Rhodamine-phalloidin according to manufacturer’s instructions (Molecular Probes). Cells were mounted on slides using Fluorescent Mounting Medium with DAPI (GBI Labs, WA, USA) and were visualized using a Leica SP5 microscope, (Buffalo Grove, IL, NA), lens × 63. Images were captured and analyzed using LAS LF lite software.

### Isolation of hepatic non- parenchymal cells for flow cytometry and sorting

Mice were perfused with 30 ml of cold PBS and the liver was excised. Livers were cut into small fragments, incubated (37 °C, 250 rpm for 45 min) with 5 ml digestion buffer [5% FCS, 0.5 mg/ml collagenase IV (Sigma-Aldrich, Rehovot, Israel, C5138-500MG) in PBS] and filtered through 200 μM wire mesh. This was followed by three cycles of washings with PBS at 400 rpm, 4 °C, 5 min, harvesting the supernatant and discarding the parenchymal cell pellet. The supernatant was centrifuged at 1500 rpm, 4 °C, 5 min and the erythrocytes in the cell pellet were lysed by a 2 min incubation with ACK Lysing buffer (Quality Biological, CAT# 118-156-721).

### Flow cytometry analysis and sorting of macrophages, monocytes and neutrophils

The following fluorescence-conjugated antibodies were used for KC characterization: APC Cy7 CD45 (BioLegend 103116), APC CD64 (BioLegend 139306), PE Cy7 CD11b (BioLegend, 101216), PE F4/80 (serotec, MCA497), FITC KI-67 (eBioscience 11-5698-80), Purified CD16/32 (BioLegend 101302), and the relevant isotype controls. KCs were defined as CD45^+^/CD11b^int^/F4/80^hi^/CD64^+^ cells^39^.

Antibodies used for liver monocyte and neutrophil characterization included: APC Cy7 CD45 (BioLegend 103116), PE F4/80 (serotec, MCA497), PE-Cy7 CD11b (BioLegend, 101216), PerCP Cy 5.5 LY6C (BioLegend, 128012), Pacific blue MHC class II (I-Ab, BioLegend 107620), Alexa Fluor® 488 CX3CR1 (BLG-149021), Purified CD16/32 (BioLegend, 101302) and the relevant isotype controls.

Antibodies used for BM and blood monocyte characterization included: APC Cy7 CD45 (BioLegend 103116), PE-Cy7 CD11b (BioLegend, 101216), Brilliant Violet 421 LY6G (BioLegend, 127627), APC CD115 (eBioscience 17-1152-82), PerCP Cy 5.5 LY6C (BioLegend, 128012), purified CD16/32 (BioLegend, 101302) and the relevant isotype controls.

Cells were analyzed on a Gallios^TM^ Flow Cytometer (Beckman Coulter) and sorted on a BD FACSAria Cell Sorter (BS Biosciences). Flow cytometry analysis was performed using Kaluza Analysis Software.

### Immunofluorescence of frozen tissue sections

Liver samples obtained from EPO or diluent-injected mice were frozen at −80 °C, sectioned, and stained for F4/80 (eBioscience, 14-4801-81) followed by a Cy3 conjugated Goat anti Rat (Jackson ImmunoResearch, CAT#112-165-167).

### Real-time quantitative PCR

Total RNA from murine liver tissue or from rat RKC-2 cells was extracted and dissolved in TRIzol Reagent (Invitrogen, Grand Island, NY, USA) according to the manufacturer’s instruction, and from sorted KCs with the GENEzol TriRNA Pure Kit (Geneaid, New Taipei City, Taiwan) according to the manufacturer’s protocol. cDNA was prepared using a high capacity cDNA reverse transcription kit (Applied Biosystems, California, USA). Real-time quantitative PCR was performed using the StepOnePlus Real-time PCR system and the KAPA SYBR FAST ABI Prism qPCR Kit (Kapabiosystems). HPRT (mouse) or TBP (rat) housekeeping genes were used as an internal standardization control. Primer sequences used for amplification were as follows: Rat EPO-R, 5′-CCACATCCGCTACGAGGT-3′ (forward) and 5′-GCCTTCCAGGACCTCCAC-3′ (reverse); Rat TBP, 5′-CCCACCAGCAGTTCAGTAGC-3′ (forward) and 5′-CAATTCTGGGTTTGATCATTCTG-3′ (reverse); Mouse EPO-R, 5′-GTCCTCATCTCGCTGTTGCT-3′ (forward) and 5′-ATGCCAGGCAGATCTTCT-3′ (reverse); Mouse MCP-1/CCL-2, 5′-TCTCTCTTCCTCCACCAC-3′ (forward) and 5′-GTGGGGCGTTAACTGCAT-3′ (reverse); Mouse CXCL2, 5′-AGTGAACTGCGCTGTCAATG-3′ (forward) and 5′-TTCAGGGTCAAGGCAAACTT-3′ (reverse); Mouse CXCL1 5′–CTTGAAGGTGTTGCCCTCAG-3′ (forward) and 5′-TCTGAACCAAGGGAGCTTCA-3′ (reverse);, Mouse CCL8, 5′-TAAGGCTCCAGTCACCTGCT-3′ (forward) and 5′-ATACCCTGCTTGGTCTGGAA-3′ (reverse); Mouse CCL5 5′-CTGCTGCTTTGCCTACCTCT-3′ (forward) and 5′-CCCACTTCTTCTCTGGGTTG-3′ (reverse); Mouse IFNγ, 5′-TCAAAAGAGTTCCTTATG-3′ (forward) and 5′-TACGAGGACGGAGAGCTG- 3′ (reverse); Mouse NOS2, 5′-ACCTTGTTCAGCTACGCC-3′ (forward) and 5′- CATTCCCAAATGTGCTTG- 3′ (reverse); Mouse IL-10, 5′-CAGAGCCACATGCTCCTA-3′ (forward) and 5′-GTCCAGCTGGTCCTTTGT-3′ (reverse); Mouse TGFβ, 5′-TGGAGCAACATGTGGAAC’−3′ (forward) and 5′-CAGCAGCCGGTTACCAAG-3′ (reverse), Mouse CX3CL1, 5′-CATGTGCGACAAGATGACCT-3′ (forward) and 5′-CAGAAGCGTCTGTGCTGTGT-3′ (reverse); Mouse HPRT, 5′-TCCTCCTCAGACCGCTTTT-3′ (forward) and 5′-CCTGGTTCATCATCGCTAATC-3′ (reverse); Mouse RPLPO, 5′-CAACCCAGCTCTGGAGAAAC-3′ (forward) and 5′-GTTCTGAGCTGGCACAGTGA-3′ (reverse).

### ELISA assay

Blood from treated or untreated mice was incubated for 2 h at 25 °C and centrifuged at 6,000 rpm, 25 °C for 30 min. Serum was collected and diluted 1:2 (for EPO or diluent-injected mice) or 1:10 (for *Tg6*-EPO overexpressing mice or wild type mice) in 96 well-plates. CCL2 protein levels were assayed by an ELISA kit (CAT #MJE00, R&D systems), according to the manufacturer’s instructions. Plates were read at 450 nm in an ELISA reader (SpectraMAX 190 microplate reader).

### Statistical analysis

Values are presented as mean ± SEM unless otherwise indicated; The non parametric Mann-Whitney U test was used for comparison. A significant difference between groups was defined as p < 0.05. All data generated or analyzed during this study are included in this published article.

## Electronic supplementary material

Supplementary Figures 1S and 2S
