# Supplementary material for: Erythropoietin enhances Kupffer cell number and activity in the challenged liver
Source: Sci Rep. 2017 Sep 4;7:10379. doi: 10.1038/s41598-017-11082-7 (PMC5583293; doi:10.1038/s41598-017-11082-7)
Supplement: Supplementary file 1 — Supplementary Figures 1S and 2S [file 41598_2017_11082_MOESM1_ESM.doc]

**Erythropoietin enhances Kupffer cell number and activity in the challenged liver**

Dafna Gilboa^1^, Yasmin Haim-Ohana^1^, Naamit Deshet-Unger^1^, Nathalie Ben-Califa^1^, Sahar Hiram-Bab^2^, Debby Reuveni^3^, Ehud Zigmond^3^, Max Gassmann^4^, Yankel Gabet^2^, Chen Varol^3*^ and Drorit Neumann^1*^

^1^Department of Cell and Developmental Biology, Sackler Faculty of Medicine, Tel Aviv University, Tel Aviv, ^2^Department of Anatomy and Anthropology, Sackler Faculty of Medicine, Tel Aviv University, Tel Aviv, ^3^The Research Center for Digestive Tract and Liver Diseases, Sourasky Medical Center and Department of Clinical Microbiology and Immunology, Sackler Faculty of Medicine, Tel Aviv University, Tel Aviv. ^4^Institute for Veterinary Physiology, Vetsuisse Faculty and Zurich Center for Integrative Human Physiology (ZIHP), University of Zurich, Zurich, Switzerland. *These authors contributed equally to this work.

**Address correspondence to:**

Drorit Neumann, Department of Cell & Developmental Biology

Tel: +972-3-6407256; Fax: +972-3-6407432; Email: histo6@post.tau.ac.il

Sackler Faculty of Medicine, Tel Aviv University

P.O. Box 39040, Tel Aviv 69978

Israel

**
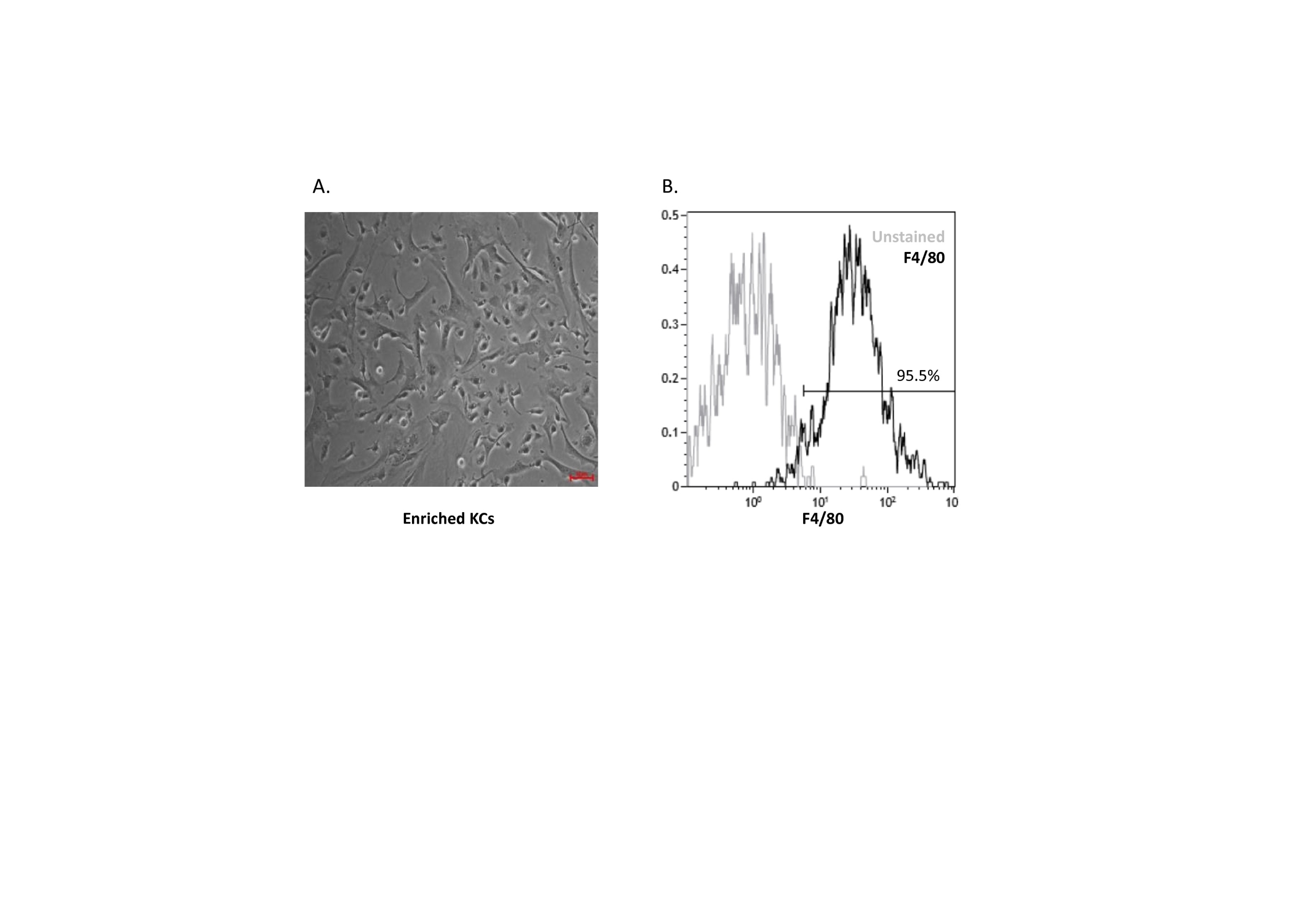
**

**Figure S1. KC population purity following enrichment and growth in culture.** Livers were harvested from C57BL/6J mice. After digestion with Collagenase type IV, parenchymal cells were discarded and KCs were isolated by selective adherence to plastic. Briefly, after incubation for 1 h, non-adherent cells were removed from the dish by gently washing with PBS and the adherent KCs were cultured for 10 days and observed under light microscopy (Nikon ECLIPSE Ti-S) (A). KCs were identified by flow cytometry using monoclonal anti-mouse F4/80 antibody [BM8.1] conjugated to APC, BG-02922-80-25 (Biogems, CA) (B).

**
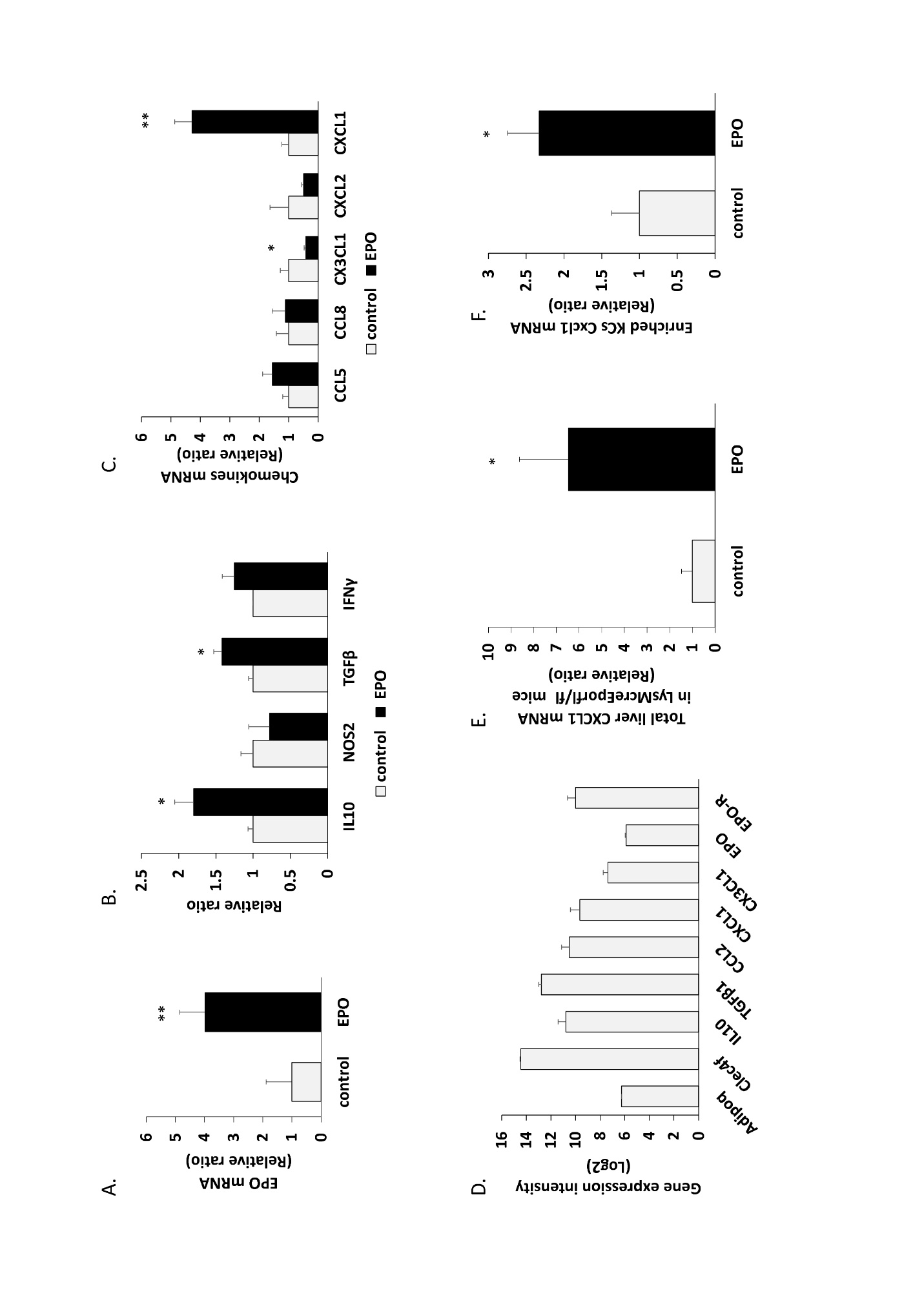
**

**Figure S2. EPO effects on the levels of gene expression of cytokines, chemokines, and EPO.** A. EPO transcript levels in total liver cells from diluent (control) or EPO-injected mice, were evaluated by RT-PCR. Graph represents EPO transcript levels ± SEM, N = 5-6, **p < 0.01. B. IL10, NOS2, TGFβ, and IFNγ cytokine transcript levels in total liver cells from diluent (control) or EPO-injected mice, were evaluated by RT-PCR. Graph represents the transcript levels ± SEM, N = 4-8, *p < 0.05. C. CCL5, CCL8, CX3CL1, CXCL2 and CXCL1 chemokine transcript levels in total liver cells from diluent (control) or EPO-injected mice, were evaluated by RT-PCR. Graph represents the transcript levels ± SEM, N = 4-9, *p < 0.05, **p < 0.01. D. The Log_2_ gene expression intensity values of selected genes in KCs sorted from steady state livers were extracted from our previously published database[^1^](#_ENREF_1). Triplicate replicates of KCs from a pool of seven mice, were sorted. Database is deposited at the National Center for Biotechnology Information Gene Expression Omnibus public database under accession number GSE55606. Raw gene expression intensity data are presented as Log_2_ values of Mean ± SD. E. *LysM^cre^-Epor^fl/fl^* mice were injected 6 times on alternating days, over 2 weeks with 180 U EPO, or diluent (control). CXCL1 transcript levels of total liver cells from *LysM^cre^-Epor^fl/fl^* mice were evaluated by RT-PCR. Graph represents CXCL1 transcript levels ± SEM, N = 4, *p < 0.05. F. CXCL1 transcript levels of enriched KCs treated with EPO or diluent (control) *in vitro* (N = 5) were evaluated by RT-PCR, graphs represent mean ± SEM, *p ≤ 0.05.

1. Zigmond, E. *et al.* Infiltrating monocyte-derived macrophages and resident kupffer cells display different ontogeny and functions in acute liver injury. *J Immunol* **193**, 344-353 (2014).
